# Chemical Manipulation of the Endosome Trafficking Machinery: Implications for Oligonucleotide Delivery

**DOI:** 10.3390/biomedicines9050512

**Published:** 2021-05-05

**Authors:** Rudolph L. Juliano

**Affiliations:** Initos Pharmaceuticals LLC, Chapel Hill, NC 27514, USA; rudyatinitos@gmail.com

**Keywords:** oligonucleotide, delivery, endosome, small molecule

## Abstract

Antisense oligonucleotides (ASOs), siRNA and splice switching oligonucleotides (SSOs) all have immense potential as therapeutic agents, potential that is now being validated as oligonucleotides enter the clinic. However, progress in oligonucleotide-based therapeutics has been limited by the difficulty in delivering these complex molecules to their sites of action in the cytosol or nucleus of cells within specific tissues. There are two aspects to the delivery problem. The first is that most types of oligonucleotides have poor uptake into non-hepatic tissues. The second is that much of the oligonucleotide that is taken up by cells is entrapped in endosomes where it is pharmacologically inert. It has become increasingly recognized that endosomal trapping is a key constraint on oligonucleotide therapeutics. Thus, many approaches have been devised to address this problem, primarily ones based on various nanoparticle technologies. However, recently an alternative approach has emerged that employs small molecules to manipulate intracellular trafficking processes so as to enhance oligonucleotide actions. This review presents the current status of this chemical biology approach to oligonucleotide delivery and seeks to point out possible paths for future development.

## 1. Introduction 

A major impediment to the therapeutic use of oligonucleotides concerns the inefficient delivery of these molecules to their intracellular sites of action within tissues [1,2,3]. There are multiple barriers to oligonucleotide delivery, but, over the last few years, it has become clear that one major issue concerns the non-productive entrapment of oligonucleotides within endomembrane compartments [4,5]. All forms of oligonucleotides enter cells by some form of endocytosis, and, typically, most of this material is retained within endosomes while only a tiny fraction reaches targets in the cytosol or nucleus. Recently, attention has focused on the late endosome/multi-vesicular body as a key site for the release of oligonucleotides from endosomal compartments. There have been many attempts to increase endosomal release; these often involve use of lipid or polymer nanoparticles [6,7,8]. In contrast, however, several recent studies have explored the use of small molecules that perturb endomembrane trafficking as a means of enhancing transfer of oligonucleotides to the cytosol. This article provides a brief overview of the intracellular trafficking of oligonucleotides and reviews the extant literature on chemical manipulation of that process. It also suggests some additional chemical biology approaches to overcome the endosomal trapping barrier to oligonucleotide delivery.

## 2. Intracellular Trafficking Processes

**Figure 1:**
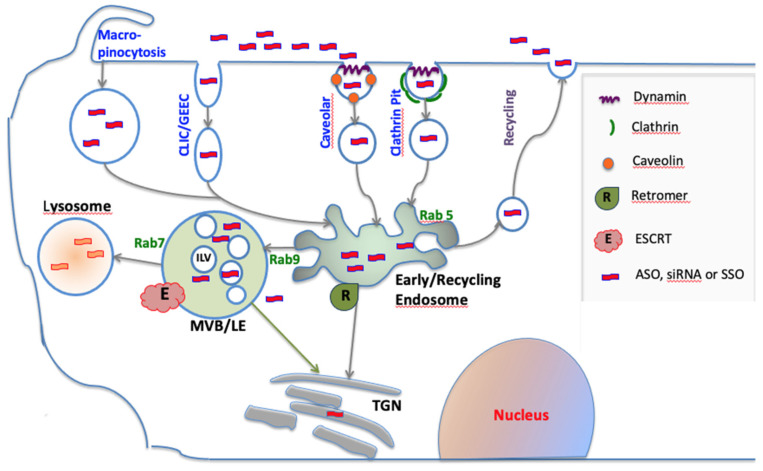
Cell uptake and trafficking of oligonucleotides. Oligonucleotides enter cells via several endocytic pathways that may depend on clathrin, caveolin or dynamin. All uptake pathways initially lead to the early/re-cycling endosome compartment. Most internalized oligonucleotide accumulates in late endosomes/multivesicular bodies (MVB/LEs) and in lysosomes; however, some trafficking to other membrane bound compartments does occur. Within endomembrane compartments, oligonucleotides are pharmacologically inert. However, a very small portion of internalized oligonucleotide can spontaneously escape to the cytosol. The endomembrane system is controlled by a plethora of proteins and protein complexes. The Rab family of GTPases regulates many aspects of trafficking, while individual members can be markers for distinct endomembrane compartments. The formation of intralumenal vesicles (ILVs) within MVBs is regulated by the multi-protein ESCRT complex. The Retromer complex may deliver oligonucleotides to the trans-Golgi instead of to lysosomes.

Cells have multiple pathways of endocytosis, including those involving clathrin- or caveolin-based vesicles, macropinocytosis and the CLIC/GEEC pathway important in fluid-phase uptake [9]. All forms of oligonucleotides, whether as free molecules, molecular conjugates, or associated with nanoparticles, utilize these pathways to varying degrees. Each of these pathways ultimately converges on early endosomes (EEs) that play a key role in the sorting of internalized materials to various subcellular destinations. The bulk of the luminal contents of EEs will traffic to downstream endomembrane compartments, initially multivesicular bodies/late endosomes, and then lysosomes [10]. However, molecules destined for export can enter the tubulations of EEs, be pinched off into small vesicles and then traffic back to the cell surface for release [11]. Similarly, EEs can also distribute materials to the trans-Golgi compartment via vesicles generated by the Retromer complex [12]. EEs mature to multivesicular bodies (MVBs); these are non-tubulated vesicles with a pH of about 5.5 that contain numerous intraluminal vesicles (ILVs) formed by inward scission of the MVB membrane through the action of the ESCRT complex [13,14]. Subsequently, MVBs transition to late endosomes (LEs) having a pH of about 5. Ultimately, LEs and their contents fuse with lysosomes (LYs), the low pH (4.5) hydrolase-rich structures that play a key role in digesting internalized materials [15]. These pathways are depicted in Figure 1.

The complex processes of intracellular trafficking are carefully orchestrated by a plethora of protein and non-protein components. A detailed description of the mechanisms involved is beyond the scope of this brief review. However, Table 1 outlines some of the key components involved.

It is important to note that the depiction of intracellular trafficking presented in Figure 1 is a major simplification of some very complex processes. Contemporary work on trafficking has shown that there are multiple trafficking pathways, that different cell surface receptors can traffic in different pathways and that this can depend on the state of receptor activation [27]. This complexity is very relevant to oligonucleotide pharmacology. For example, it has been shown that lipid conjugated siRNAs traffic through pathways similar to those used by EGF receptor but not those used by transferrin receptor [28]. Similarly, it has been shown that the initial uptake route can influence the pharmacological efficacy of oligonucleotides [29]. The concept of “productive” versus “non-productive” oligonucleotide uptake has become fairly well established [4,30,31]. However, as discussed below, we are only beginning to delineate the productive uptake process. Precise quantitative analysis of the relationships between oligonucleotide uptake, subcellular distribution and ultimate biological effect remains a challenging task, as described in a recent review [32]. 

## 3. Oligonucleotide Release from Endosomes

Recently, several diverse studies have led to a conceptual convergence implicating an intermediate endomembrane compartment, likely MVBs, as a key site for productive oligonucleotide escape to the cytosol [4,33]. An early but elegant study used advanced confocal microscopy, as well as molecular techniques, to manipulate trafficking and followed the fate of siRNA delivered via cationic lipid nanoparticles. The siRNA progressed from EEs, to LEs, to LYs will the bulk of the material (~98%) remaining within the endomembrane compartments. The modest amount of siRNA that escaped to the cytosol did so from an early/intermediate compartment before transport to LEs or LYs [34]. Another study also used sophisticated microscopy to follow the fate of siRNA delivered to cells in large liposomes. Here endosomes were observed to release siRNA via a burst mechanism, and, while the late endosome marker Rab7 was present during the burst, the EE marked, Rab 5 was not, once again indicating release from an intermediate compartment [35]. 

Studies on the intracellular fate of “free” oligonucleotides have come to similar conclusions about the key role of an intermediate endosomal compartments. Thus, one study, which is further discussed below, used small molecules termed OECs to promote oligonucleotide escape from endosomes. This study demonstrated that effective concentrations of the OECs did not affect early endosomes or lysosomes but rather an intermediate compartment [36]. As part of a series of studies on factors that affect oligonucleotide efficacy that are discussed in more detail below, Crooke and colleagues showed that perturbation of certain MVB lipids or proteins significantly influenced oligonucleotide activity [37,38]. A recent study examined the effect of lysosomotropic drugs, such as chloroquine on the intracellular distribution of siRNA [39]. In this case, both late endosomes and lysosomes seemed to be involved in the process. Thus, the preponderance of evidence suggests a key role for intermediate endosomal compartments, likely MVBs or LEs, in the productive release of either free or liposomal oligonucleotides to the cytosol. 

An important aspect of oligonucleotide release is that this process is accompanied by damage to the endosomal membranes. Galectins are endogenous beta-galactoside binding lectins that rapidly relocate from the cytosol to damaged intracellular membranes and thus can serve as sensors for membrane damage [40]. Several studies have used either anti-galectin antibodies or galectin-chimeras with fluorescent proteins to document galectin association with endosomes during release of oligonucleotides [35,39,41]. Whether this damage is associated with significant toxicity to cells is unclear at this point.

## 4. Influencing Oligonucleotide Actions by Manipulation of the Endosomal Trafficking Machinery 

A number of investigators have sought to influence the pharmacological actions of oligonucleotides my manipulating components of the endosomal machinery. A leading example of this comes from the work of Crooke and colleagues at Ionis Pharmaceuticals who have extensively studied the roles of multiple proteins in the actions of phosphorothioate antisense oligonucleotides (ASOs) [42]. Included among these studies are several that specifically address proteins involved in endomembrane trafficking [43,44,45,46]. These studies generally utilized three approaches: (i) identification of cell lysate proteins that bound to ASOs; (ii) co-localization of proteins with ASOs via fluorescence microscopy; and (iii) perturbation of protein levels, using siRNA, followed by evaluation of the effect on ASO activity. One study showed that siRNA mediated depletion of the early endosome protein EEA1 or of Rab5C or Rab7A reduced the effectiveness of ASOs [46]. Another study examined proteins involved in ER-Golgi transport. Surprisingly, siRNA-mediated reduction of COPII Golgi coat proteins reduced ASO activity by causing slower release from LEs [44]. Further studies showed that siRNA-mediated reduction of M6PR, which is involved in Golgi to LE transport, as well as of GCC2, a tethering protein, could both reduce ASO effectiveness [45]. Another interesting observation from this group concerns lysobisphosphatidic acid (LBPA), a lipid that is preferentially found in ILVs. Treatment of cells with an anti-LBPA antibody reduced ASO effectiveness, thus emphasizing the role of ILVs in ASO trafficking [38]. Another group of investigators at Ionis found that autophagosomes were involved in ASO processing and that enhancing autophagy increased ASO effects [47]. One point to keep in mind concerning these various studies is that the observed effects were rather modest. Thus, none of the factors identified seem to play an all or none role in ASO effectiveness but rather contribute to the process. Another group used a shRNA screen to identify TSG101, a component of the ESCRT complex, as a factor in oligonucleotide trafficking [48]; however, this work has not been followed up. Thus, despite some limitations, the overall context provided by these various studies is that components of the endosomal trafficking machinery are intimately involved in the subcellular fate and thus the pharmacological effectiveness of oligonucleotides. 

## 5. Manipulation of Oligonucleotide Delivery and Effect Using Small Molecules

Following this context, it is a natural evolution to think about using small molecules to manipulate the endosomal system with the intent of enhancing oligonucleotide actions. It has been known for decades that lysosomotropic compounds such as chloroquine can promote the escape of large molecules from the endosomal system. This has usually been attributed to the “proton sponge effect”, whereby the lysosomotropic drugs are protonated and trapped within endosomal compartments, leading to an influx of water molecules and swelling and disruption of the compartment [49]. 

More recently, several groups have more systematically approached the strategy of using small molecules to enhance oligonucleotide effects, either by focusing on individual molecules or by undertaking screening of chemical libraries. One study screened a small library of drug-like molecules for their ability to improve the efficacy of a chemically modified siRNA in cells [50]. A drug called Guanabenz had this effect in the 50 uM range; however, further investigation showed that the drug affected uptake of the siRNA rather than release from endosomes. Another study screened approximately 45,000 compounds for their effects on siRNA administered to cells either in lipid nanoparticles (LNPs) or as cholesterol conjugates; the target in this case was a GFP reporter and screening was done by fluorescence microscopy [51]. A number of compounds were identified that either improved uptake or increased endosome escape; the set of compounds that acted via endosome escape seemed to do so by several different mechanisms but generally had only modest effects. Utilizing a different strategy, investigators screened a small molecule library for effects on the activity of antagomirs that regulated an EGFP reporter in cells [52]. One compound, 6BIO, seemed of particular interest since it also downregulated androgen receptor in prostate cancer cells. However, whether 6BIO affects endosomal trafficking is unclear at this point. In another small-scale screening study, an FDA-approved drug that affects muscle ryanodine receptors, Dantroline, was reported to increase the effectiveness of oligonucleotides that correct a splicing defect in a murine Duchenne muscular dystrophy model [53]. More recent studies have extended these findings to other drugs that affect ryanodine receptors and to additional dystrophic mutations [54]; however, the exact mechanism by which these drugs affect oligonucleotides remains undefined. In the same therapeutic context, other studies have utilized aminoglycosides [55], hexose [56] or saponins [57] to enhance the effect of splice switching oligonucleotides in cell and murine models of Duchenne dystrophy. In the case of the aminoglycosides the authors attributed the effect to enhanced delivery, while the precise mechanism is unclear in the case of hexose. In the case of the detergent-like saponins, altered membrane permeability seems a likely mechanism. In a very different vein, Gooding et al. [58] designed strongly cationic small molecules that bind to siRNA and enhance its uptake and effects. This approach seems intermediate between typical cationic lipid or polymer transfection methods and the use of soluble monomeric small molecules to affect oligonucleotide actions.

Over the last few years, my laboratory has pursued a series of studies on small molecules that enhance oligonucleotide actions. We designate such compounds OECs (oligonucleotide enhancing compounds), a term that is not very imaginative but is one that clearly describes the actions of these molecules. Our interest in this aspect grew out of previous work from us and from others on receptor-targeted versus non-targeted delivery of oligonucleotides [31,59,60,61,62]. It became apparent from that work that the uptake path and subsequent intracellular trafficking played an important role in oligonucleotide pharmacology and thus that it was worth looking for molecules that perturbed trafficking processes [29,31]. 

The first OEC we identified was a compound named Retro-1. This compound had emerged from a screen for molecules that inhibited the actions of toxins. Investigations showed that Retro compounds blocked the trafficking of certain plant and bacterial toxins at the step of retrograde transfer from early endosomes to trans-Golgi [63,64]. In collaboration with Professor D. Gillet, we tested Retro-1 for its ability to influence oligonucleotide actions. We primarily used splice switching oligonucleotides (SSOs) with a 2’-O-methyl phosphorothioate chemistry and cell lines stably transfected with luciferase or EGFP reporters whose expression could be upregulated by correction of a splicing defect [65]. These studies showed that Retro-1 in the 20–100 uM concentration range could substantially enhance SSO effects. This was associated with a partial transfer of fluorophore-labeled SSOs from cytosolic vesicles to the nucleus. We also showed that Retro-1 had no effect on lysosomal pH, thus distinguishing this compound from lysosomotropic compounds such as chloroquine. Studies of the effect of Retro-1 on the co-localization of fluorophore-labeled oligonucleotides with protein markers of various endosomal compartments revealed that Retro-1 affected Rab7/9 positive late endosomes rather than Lamp-1 positive lysosomes. In a subsequent study, we examined the effects of several Retro-1 analogs on oligonucleotide actions and on toxin trafficking [66]. We identified analogs that affected toxins but not oligonucleotides and vice versa, while Retro-1 was the only compound that significantly affected both. This indicates that the molecular target(s) involved in increasing oligonucleotide actions and those involved in blockade of toxin trafficking are distinct. In another study, in collaboration with Professor A. Grandas, we evaluated the effects of Retro-1 that was directly conjugated to SSOs [67]. Unfortunately, there was no enhancement of SSO action by this means. Although Retro-1 remains an interesting molecule, there are clearly limitations to its therapeutic use. Thus, it requires rather high concentrations to have an effect and that effect is less than that achievable with, for example, cationic lipid transfection. Additionally, our attempt to use Retro-1 to correct splicing in a murine model had only limited success. Thus, we began to pursue additional OECs. 

We screened > 100,000 compounds in a 384 well format, using cells that had a luciferase reporter with a splicing defect and SSOs designed to correct that defect [68]. Cells were pre-incubated with 100 nM SSO overnight and then exposed to 25 uM test compound for 5 h, followed by harvesting and analysis of luciferase induction. Cells receiving 300 uM chloroquine were used as a positive control, while cells receiving diluent were negative controls. Library compounds that produced an induction 50% that of the positive control were considered positive in the initial screen and were further characterized. The hit rate in this screen was rather low (0.04%), but, ultimately, two distinct families of compounds were identified that satisfied the following criteria: (i) they strongly increased luciferase induction by the SSO but not with a mismatched oligonucleotide, and (ii) they were not toxic at concentrations needed to substantially increase induction. The prototype compound of a series of 3-deazapteridine analog OECs is termed UNC7938 [68] while the prototype of a series of benzimidazole OECs is termed UNC2383 [69]. The newly identified OECs were substantially more effective than Retro-1; for example, 20 uM UNC7938 provided a 220-fold increase in luciferase induction versus 11-fold at 100 uM Retro-1. In addition to SSOs, both the UNC7938 and UNC2383 compounds could enhance the actions of antisense oligonucleotides and siRNAs. An investigation of mechanism demonstrated that UNC7938 treatment resulted in a partial shift of fluorophore-tagged SSO from cytosolic vesicles to the nucleus similar to the case of Retro-1. Moreover, similar was the observation that UNC7938 primarily affected co-localization of oligonucleotides with the late endosome marker Rab7 rather than the lysosome marker Lamp-1 (it should be noted, however, that there are not precise delineations between the various endomembrane sub-compartments). With both UNC7938 and UNC2383, a strong enhancement of SSO effects could be obtained at concentrations that had little effect on lysosomal pH, thus emphasizing the distinction between the OECs and typical lysosomotropic compounds. 

In contrast to the case with Retro-1, we were able to obtain significant in vivo effects with both families of new OECs. Here we used a murine transgenic reporter model that had an EGFP cassette with a splicing defect; successful in vivo delivery of a SSO corrects the defect and allows expression of EGFP mRNA and protein [70]. Using this model, we demonstrated that the SSO+OEC combination could attain partial correction of splicing in several extra-hepatic tissues including lung, intestine heart and kidney [68,69]. 

Subsequently we have pursued initial structure–activity studies with the UNC7938 series and have defined the essential features that contribute to activity [36]. This study also confirmed the concept that OEC UNC7938 acts at an intermediate compartment in the endosomal trafficking system. Thus, if oligonucleotides were forced to be retained in early endosomes there was no effect of UNC7938; additionally, effective concentrations of this compound clearly failed to reduce lysosomal pH. Thus, the OEC seems to act at a site in the trafficking pathway that is after early endosomes, but before lysosomes, probably within the key MVB/LE compartments that are the natural sites of productive oligonucleotide release to the cytosol [4,33]. Additional reinforcement regarding the site of action of OEC UNC7938 comes from a recent study that demonstrated that this compound binds preferentially to lysobisphosphatidic acid, a lipid found predominately in MVBs [71]. Thus, the UNC7938 and UNC2383 OECs have many interesting and valuable characteristics as modulators of oligonucleotide release from endosomes and are currently being explored for possible therapeutic use. A possible limitation, however, is that there is a rather narrow window between effective and toxic doses for these compounds. Hopefully further medicinal chemistry development will help to open that window. In summary, while there has been significant progress in using small molecules to enhance oligonucleotide actions there is clearly much that remains to be done. 

## 6. Possible Chemical Interventions in Oligonucleotide Trafficking

The final section of this review will explore new possibilities regarding small molecule manipulation of oligonucleotide trafficking. A key strategy for seeking additional molecules would be to extend the chemical space evaluated by screening libraries that are larger than and/or different from those already used. One note of caution, however, is that it might be wise to exclude lipophilic amines from such screening, since molecules of this type are likely to resemble typical lysosomotropic agents like chloroquine and thus would not represent functionally novel entities. A second consideration would be the nature of the screening assay. Screens where a “hit” results in a positive outcome, such as increased expression of an endogenous or reporter protein, provide advantages over screens where a hit results in an inhibition. In the latter case it is difficult to discriminate between a true hit and simple cytotoxicity. Positive screens have already proven their worth in identifying compounds that modulate oligonucleotide pharmacology [52,68]. 

In addition to non-selective screening, it would be important to focus on molecules that affect key steps in the intracellular trafficking pathways used by oligonucleotides. As mentioned above, several studies have implicated late endosomes/multi-vesicular bodies and possibly the trans-Golgi as key sites for oligonucleotide release to the cytosol [4,36,38,44]. The detailed mechanisms underlying preferential oligonucleotide release from these sites remains unclear. However, one might note that while the entire endomembrane trafficking machinery is dynamic, these sites are particularly active in terms of the formation, scission and fusion of vesicles. As discussed in more detail elsewhere [33], regions of active membrane dynamics are likely to be particularly permeable, thus potentially permitting release of endosomal contents including oligonucleotides.

Regrettably, the inventory of small molecules that affect endosomal trafficking is rather limited, as indicated in a comprehensive review of the topic published several years ago [72]. While there are a number of inhibitors of the initial steps of endocytosis, none of these is likely to enhance oligonucleotide trafficking. Of more interest are compounds that affect later aspects of trafficking. Several of these are described in the abovementioned review, including the Retro compounds, Brefeldin A, Vacuolin-1, Exo1,2 and cl-976. Table 2 updates the information on these compounds, as well as mentioning additional relevant small molecules. The compounds are grouped according to their proposed actions on particular subcellular compartments or trafficking components. However, as with most small molecule drugs, these compounds undoubtedly have multiple targets in cells. With the exception of the Retro compounds, there are no published reports indicating that the molecules listed in Table 2 have any effect of oligonucleotide actions. Nonetheless, in light of their proposed cellular effects, it may be worthwhile to test some of these molecules for the ability to enhance oligonucleotide actions by modifying intracellular trafficking.

## 7. Conclusions

The intracellular trafficking of oligonucleotides is a key aspect in determining their pharmacological effects and therapeutic utilization. The trafficking machinery is highly complex, involving a plethora of protein and non-protein components. However, this very complexity offers opportunities to manipulate trafficking in ways that are advantageous to oligonucleotide pharmacology. Chemical biology strategies for enhancing oligonucleotide actions are still in their infancy, but, seemingly, there are manifold possibilities for maturation of this approach.

## Figures and Tables

**Table 1:** Components Involved in Endosomal Trafficking.

Identify of Component	Function in Trafficking	Reference
Adaptor and coat proteins	Involved in initial pinching off of membrane vesicles. A well-known example is the clathrin/dynamin system.	[16]
Tethering proteins	Provide recognition between two membrane compartments. An example are the Golgins that direct vesicles to distinct Golgi sub-compartments.	[17,18]
SNARES/NSF-SNAP	Fusion of intracellular membranes is mediated by SNARE proteins while re-segregation of SNARES is mediated by the NSF/SNAP complex.	[19,20]
ESCRT Complex	A multi-protein complex that is responsible for the generation of the ILVs that populate MVBs. This complex also plays a role in several other cellular functions.	[13,21]
Retromer complex	A multi-protein complex that forms vesicles that shuttle between early endosomes and the trans-Golgi.	[12,22,23]
Rab proteins	Members of this large family of GTPases direct many aspects of intracellular trafficking.	[24,25]
Lipids: lysobisphosphatidic acid, certain phosphatidyl inositides	These lipids are preferentially found in MVBs/LEs.	[26]

**Table 2:** Small Molecules That May Affect Oligonucleotide Trafficking.

Endo- Membrane Target	Small Molecule	Mechanism of Action	Reference
**GOLGI**			
	Secramine	A CDC42 inhibitor that affects export from the Golgi. Likely multiple effects on cytoskeleton.	[73]
	YM201636	A PI-3P-5-kinase inhibitor that blocks endosome to trans-Golgi traffic, possibly endosome to lysosome traffic, and is a modulator of autophagy.	[74,75]
	A5, others	Small molecule inhibitors of traffic between the trans-Golgi and endosomes.	[76]
	CI-976	A lysophospholipid acyltransferases inhibitor that blocks a late step in COPII vesicle formation in Golgi.	[77]
	Brefeldins	Inhibit the GEFS of Arf GTPases resulting in Golgi disassembly.	[78,79]
**LE/MVBs**			
	Vacuolins	Induce the formation of large, swollen structures derived from endosomes and lysosomes; vacuolins are thought to be inhibitors of PI-3P-5-kinase.	[80,81,82]
	Ceramide	Promotes budding of intraluminal vesicles.	[83]
**Retromer**			
	Retro compounds	Block toxin trafficking, increase effects of oligonucleotides; mechanism involves inhibition of endoplasmic reticulum exit site component Sec16A.	[63,64,65,66]
**RABs**			
	ABMA DABMA	Adamantane like compounds reduce expression of Rab7a and delay intracellular trafficking of endosomal contents.	[84]
	CID 1067700	Partially selective inhibitor of Rab 7.	[85]
	Peptide inhibitor	Rab 8a inhibitor.	[86]
	Statins	Inhibit Rab associations with Endosomes.	[87]

## Data Availability

Not applicable.
